# Exploring T Cell and NK Cell Involvement in Ankylosing Spondylitis Through Single‐Cell Sequencing

**DOI:** 10.1111/jcmm.70206

**Published:** 2024-12-16

**Authors:** Tianyou Chen, Shengyu Ning, Jichong Zhu, Xinli Zhan, Chenxing Zhou, Chengqian Huang, Shaofeng Wu, Bin Zhang, Sitan Feng, Jiarui Chen, Jiang Xue, Zhenwei Yang, Chong Liu

**Affiliations:** ^1^ The First Affiliated Hospital of Guangxi Medical University Nanning Guangxi People's Republic of China

**Keywords:** ankylosing spondylitis, cell communication, immune cell, immune regulation, single‐cell sequencing

## Abstract

To uncover the complex immune mechanisms driving inflammation in ankylosing spondylitis and lay the groundwork for identifying new therapeutic targets and innovative approaches, we conducted 10× single‐cell sequencing on bone marrow cell samples collected from the vertebrae of three AS patients and three non‐AS patients. Using single‐cell sequencing data, we analysed the expression of differentially expressed genes (DEGs) by comparing AS patients with non‐AS patients. Key genes among the related DEGs were identified through protein–protein interaction networks and hub gene screening and further validated using immunohistochemistry. We performed clustering and annotation of the single‐cell sequencing data and externally validated the findings using the GSE232131 single‐cell dataset. By integrating transcriptome data, we assessed the differential expression of immune cells in AS. Finally, we explored the interactions between immune cells in AS through cell communication analysis. The upregulated gene CD74 was identified as a hub gene in T cells in AS. Further research revealed the important relationship between T cells and NK cells in the fundamental processes of AS. Additionally, we found that the macrophage migration inhibitory factor signalling pathway is prominently expressed in the interactions among various cell types in AS.

## 1 Introduction

Axial spondyloarthritis (axSpA) is a chronic inflammatory rheumatic disease characterised by manifestations in the axial, peripheral and nonarticular domains [1, 2]. It encompasses axial features such as spondylitis and sacroiliitis; peripheral manifestations such as oligoarthritis, dactylitis and enthesitis; and nonarticular manifestations such as psoriasis, uveitis and inflammatory bowel disease (IBD) [3, 4]. Axial spondyloarthritis (axSpA) is further classified into nonradiographic axSpA (nr‐axSpA) in the early stage [5] and ankylosing spondylitis (AS), which is diagnosed on the basis of radiographic sacroiliitis meeting the modified New York criteria for AS [6]. As a chronic inflammatory disorder, AS [7] is characterised by inflammation in the axial skeleton and sacroiliac joints, often impairing spinal mobility and function and significantly impacting patients' quality of life.

Ankylosing spondylitis is a disease marked by chronic inflammation and immune system abnormalities; immune regulation plays a crucial role. Recent advancements have been made in understanding the molecular mechanisms and clinical diagnosis of AS [8, 9]. Research has found that T cells are crucial for initiating and regulating immune responses to infections and play an important role in the progression of AS. Both CD4+ and CD8+ T cells are activated in AS, and they possess unique regulatory functions within the immune system [10]. Th17 cells, in particular, secrete pro‐inflammatory factors like IL‐17, which drive the inflammatory processes associated with AS. A key objective of immune regulation is to restore balance among these cells by inhibiting the overactivation of Th17 cells while increasing the proportion of regulatory T cells (Tregs) to foster immune tolerance. The increased levels of pro‐inflammatory cytokines, including TNF‐α, IL‐1, IL‐6 and IL‐17, are linked to the disease's inflammatory activity [11]. In AS, interactions among various immune cells, such as T cells, B cells, macrophages and dendritic cells, play a vital role in immune regulation. These interactions can take place through signalling pathways like NF‐κB and JAK–STAT, resulting in the overexpression of pro‐inflammatory factors [12, 13]. In addition, the integration of artificial intelligence into the medical field has further advanced our understanding of AS [14, 15]. Traditional sequencing methods provide an average assessment of genomes or transcriptomes across multiple cells, offering limited insights into cell heterogeneity [16]. In contrast, single‐cell sequencing technology [17] has emerged as a powerful tool for detecting heterogeneity among individual cells, identifying rare cell populations and facilitating detailed cell map construction. The introduction of single‐cell sequencing has significantly enhanced research in various diseases.

**FIGURE 1:**
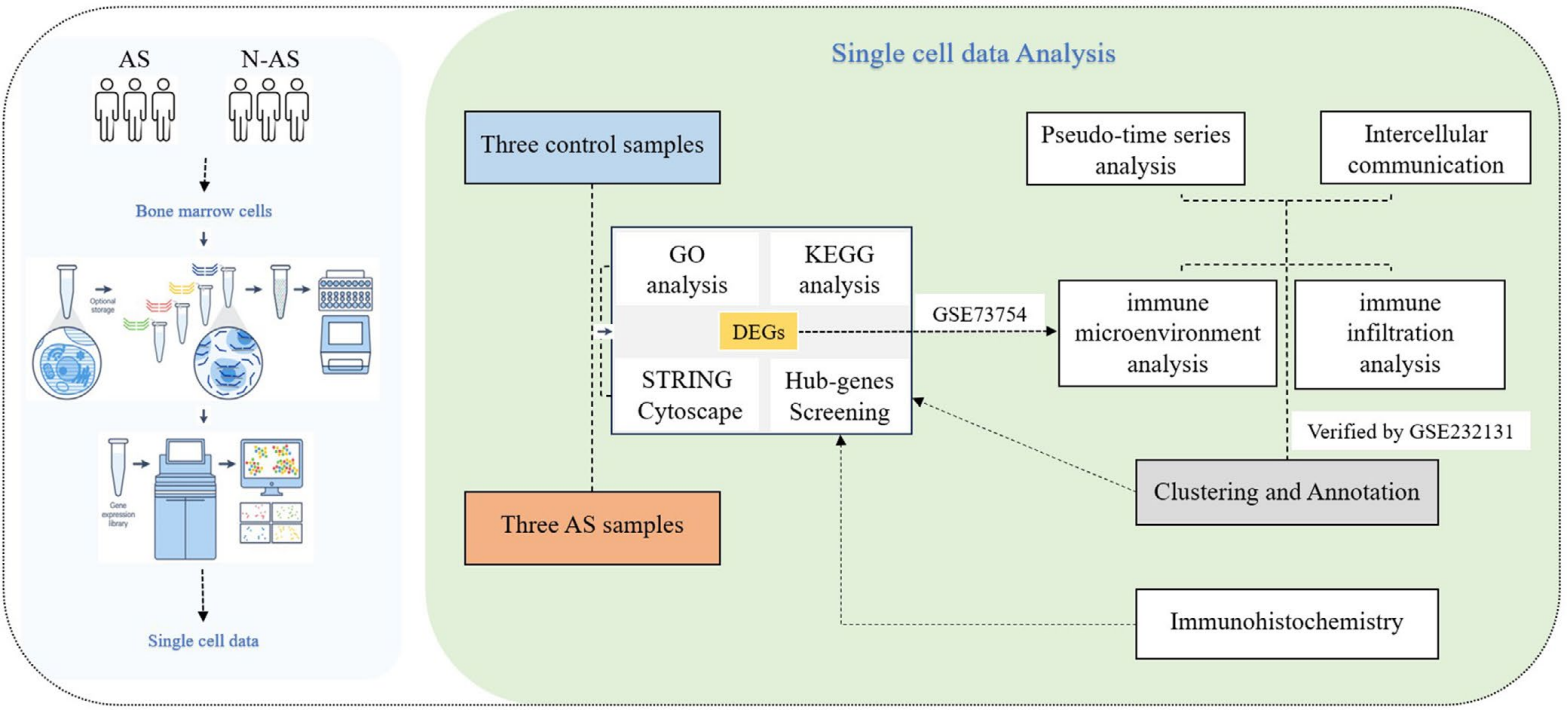
Study flowchart.

In the preliminary stages of AS research, substantial groundwork has been performed [18, 19, 20]. We further explored the intricate interactions among various immune cells during the pathological progression of AS, an inflammatory disease. Herein, we employed single‐cell sequencing to delve more comprehensively into the immunomodulatory dynamics of AS, unravelling intricate cell‐to‐cell communication relationships and identifying additional key genes with potential as therapeutic targets, thereby increasing the number of interventions under consideration. The research process is shown in Figure 1.

## 2 Materials and Methods

### 2.1 Data

This retrospective inquiry strictly followed the principles outlined in the Declaration of Helsinki, with participants providing informed consent. Ethical approval was secured from the Institutional Review Board of our institution (Approval No. 2024‐E041‐01).

#### 2.1.1 Datasets

The raw data were sourced from the GEO database to obtain comprehensive gene expression profiles. The search criteria in the GEO database specifically focused on RNA‐seq profiles related to ‘ankylosing spondylitis’. Stringent filtering measures were applied to ensure data accuracy. We obtained the GSE73754 transcriptome dataset and the GSE232131 single‐cell dataset for further analysis. For the GSE232131 single‐cell dataset, two untreated AS samples, GSM7316123 and GSM7316126, were used.

#### 2.1.2 Sample Preparation for Single‐Cell Sequencing

The samples for single‐cell sequencing were derived from bone marrow cells extracted during spinal surgery. The patient cohort included three individuals diagnosed with AS and three with thoracic/lumbar fractures. For details on sample processing, single‐cell library preparation and handling of sequencing data, consult the Supporting Information.

### 2.2 Differentially Expressed Genes Screened and Enrichment Analysis

We meticulously identified differentially expressed genes (DEGs) using the Limma R package in the single‐cell sequencing data of both the AS and non‐AS samples. The selection criterion for DEGs was a *p*‐adjusted value of < 0.05. To comprehensively understand the gene datasets, we employed Gene Ontology (GO) enrichment analysis, a widely used bioinformatics approach. Furthermore, we conducted a KEGG pathway enrichment analysis to gain deeper insights into biological mechanisms and functions.

### 2.3 Protein–Protein Interaction Network Construction and Analysis

The Search Tool for the Retrieval of Interacting Genes (STRING; [http://string‐db.org]) version 11.0 serves as a robust tool for exploring protein interactions. This functionality enables the construction of a protein–protein interaction (PPI) network characterised by intricate regulatory connections. Interactions with a combined score exceeding 0.4 were deemed statistically significant. To visualise this PPI network, Cytoscape (version 3.7.2, [http://www.cytoscape.org]) was used. Four commonly used algorithms in CytoHubba—radiality, degree, maximal clique centrality (MCC) and maximum neighbourhood component (MNC)—were employed in Cytoscape to identify the top 10 hub genes from each algorithm [21, 22]. The final hub genes were determined by taking the intersection of these results. Radiality is a centrality metric that quantifies the distance between a node and all other nodes in the network. Degree, one of the most fundamental centrality measures, indicates the number of nodes directly connected to a given node, reflecting its level of activity in the network. MCC, a distinctive algorithm within CytoHubba, identifies key nodes by evaluating the maximum clique that each node belongs to. MNC assesses centrality by calculating the largest neighbouring component of a node. By utilising multiple algorithms and combining their results to filter hub genes, this approach aims to enhance the robustness of the findings and improve the accuracy of key node identification.

### 2.4 Insights From Single‐Cell Sequencing

We utilised the Seurat package (version 3.1.4) for essential tasks such as cell normalisation and regression on the basis of the expression table. This normalisation accounted for individual sample UMI counts and mitochondrial content, producing scaled data. To address potential batch effects from sample processing and sequencing, we applied mutual nearest neighbour (MNN) correction. After this correction, we performed UMAP dimensionality reduction using the top principal components. A resolution parameter of 0.8 enabled unsupervised clustering of cells through a graph‐based method. We identified marker genes using the ‘Find All Markers’ function with the Wilcoxon rank‐sum test, applying the criteria of log fold change (log FC) > 1, *p*‐value < 0.05 and a minimum percentage (min. pct) > 0.1. For a deeper understanding of cell types, we reanalysed clusters of the same type via UMAP and additional clustering and marker analysis. Additionally, we employed the ‘Monocle’ R package for pseudo‐time series analysis to explore cell trajectories and their developmental sequences. Finally, the GSE232131 single‐cell dataset was used for external validation.

### 2.5 Immunohistochemistry

The investigation included individuals diagnosed with AS in the experimental group from the hospital, while interspinous ligaments procured from four non‐AS individuals undergoing spinal surgery constituted the control group. The basic information of the patients included in the immunohistochemistry analysis is described in Table S4. Immunohistochemistry was used to study the variations in the expression of the hub genes between the AS and control groups. The interspinous ligament tissue underwent meticulous processing, resulting in the successful acquisition of 32 immunohistochemical sections. This intricate process involves steps such as wax sealing, sectioning, antigen repair, antibody hybridisation, colour development and tissue sealing. The samples were subsequently studied under a microscope, and images of both the AS and normal control groups were captured. The positive evaluation of all immunohistochemical images was conducted using ImageJ software, and the expression differences were statistically analysed using a two‐tailed *t*‐test.

### 2.6 Expression of Hub Genes in Single‐Cell Clusters

Following the clustering, annotation and visualisation of cell types, the hub genes extracted from the PPI network were carefully identified. The expression profiles of these hub genes were subsequently comprehensively evaluated within diverse cell types.

### 2.7 Analysis of Typing Genes and Infiltration of Immune Cells

To investigate the biological differences between branches in AS pseudo‐timing analysis, we performed gene subtype analysis of branch‐specific genes using the ‘Consensus Cluster Plus’ R package combined with the GSE73754 dataset. The R software package CIBERSORT, an algorithm designed for immune infiltration deconvolution based on gene expression data, was used for immune infiltration deconvolution of distinct genotyped genes. The algorithm provided *p* values for the deconvolution of each sample, indicating the confidence level of the outcome, with a significance threshold set at *p* < 0.05 for accuracy. The CIBERSORT results were visualised via R packages, including corplot, vioplot and ggplot2.

### 2.8 Cell Communication Analysis

This study elaborates on the use of cell communication analysis, a methodology applied to elucidate interactions within single‐cell datasets, particularly in the context of single‐cell RNA sequencing (scRNA‐seq). The AS‐related study specifically employed the Monocle R package and cell‐Chat R package for pseudo‐time series analysis and cell communication analysis.

### 2.9 Statistical Analysis

Since single‐cell data generally do not satisfy the assumption of a normal distribution, after batch effect correction and dimensionality reduction processing using Harmony, we chose to conduct further analysis through non‐parametric tests. This method can effectively evaluate the differences between different cell populations and avoid the analytical bias caused by data not conforming to normality. All the statistical analyses and graphical representations were generated using R software (version 4.2.3). A *p*‐value < 0.05 indicated statistical significance.

## 3 Results

### 3.1 A Variety of Immune Cells Are Involved in AS

**FIGURE 2:**
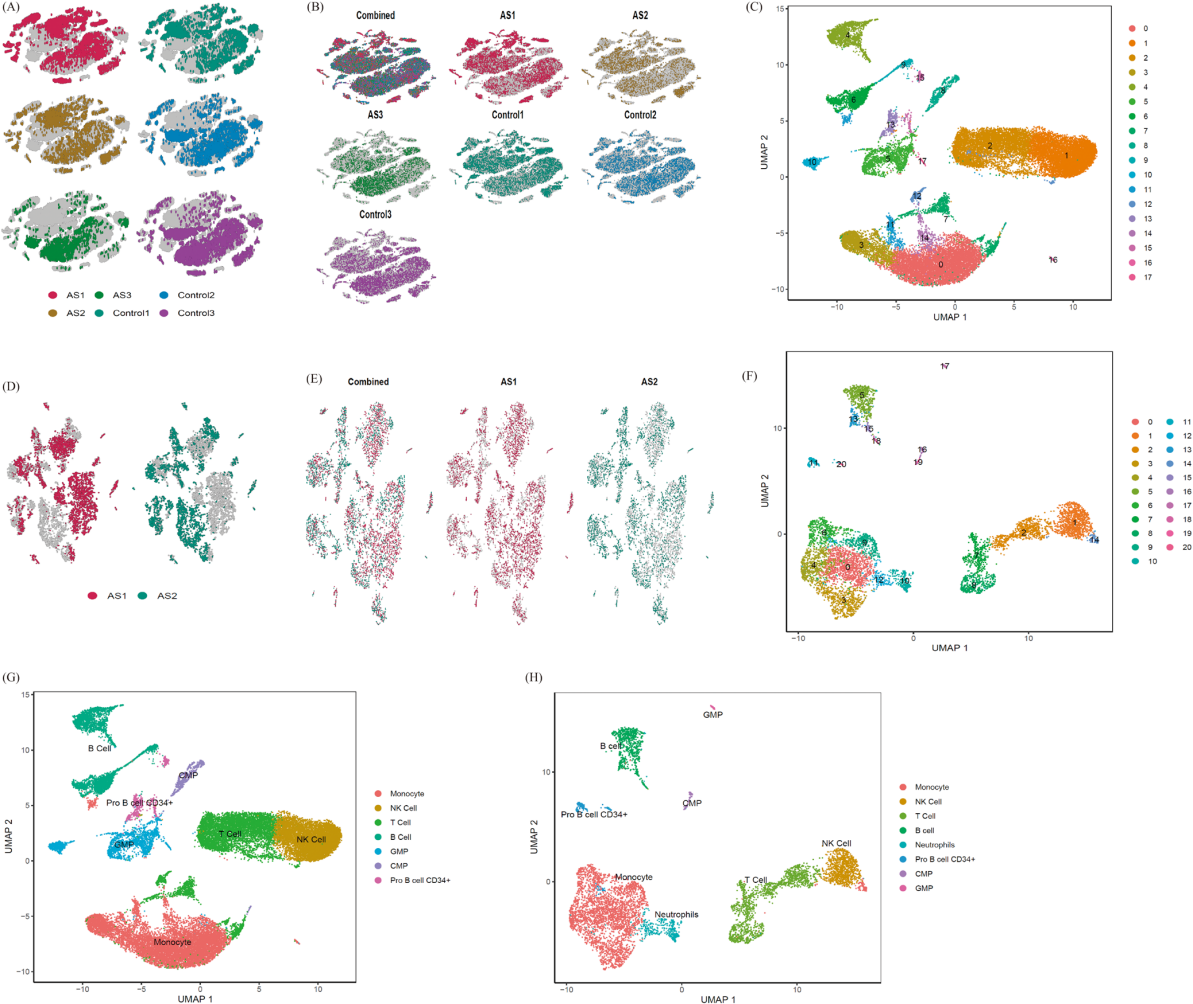
Clustering and cell‐type identification in AS and control samples. (A) Initial observation data from three AS and three control samples. (B) Observation data of six samples after batch correction using Harmony. (C) Eighteen clusters identified in the experimental group. (D) Initial observation data from two AS samples in the validation group. (E) Observation data from two AS samples in the experimental group. (F) Twenty‐one clusters identified in the validation group. (G) Seven cell types identified in the experimental group after annotation. (H) Eight cell types identified in the validation group after annotation.

In the 3V3 single‐cell experimental group, the raw data were filtered and normalised using Harmony (Figure S1A,B). Figure 2A,B illustrates the effect of de‐batching the data via Harmony, resulting in 18 clusters identified through UMAP (Figure 2C). The same approach was applied to the GSE232131 dataset, yielding 21 clusters (Figure S1C,D, Figure 2D–F). Cell‐type annotation for each cluster was conducted using the ‘SingleR’ R package. In the experimental group, seven cell types were identified, including monocytes, T cells, GMP cells, NK cells, B cells, CMP cells and Pro B cells CD34+ (Figure 2G). In the GSE232131 dataset (validation group), eight cell subsets were identified: monocytes, T cells, GMP cells, NK cells, B cells, CMP cells, neutrophils and Pro B cells CD34+ (Figure 2H). Heatmaps showing the top five DEGs for each cell type in both the experimental group and GSE232131 are displayed in Figure S1E,F, respectively.

### 3.2 Differential Genes Play a Significant Role in AS

A total of 126 DEGs were discerned from the single‐cell sequencing data when the AS group was compared with the non‐AS group. Within this set, 55 genes were upregulated, whereas 71 were downregulated (Table S1). To unravel AS‐related functional annotation and pathway enrichment, the DEGs were screened with an absolute *p* adj. value threshold of < 0.05, paving the way for subsequent GO and KEGG analyses. The comprehensive findings are detailed in Tables S2 and S3. In the context of the GO analysis (Figure S2A,B), the most enriched biological processes (BPs) included the regulation of hemopoiesis, myeloid cell differentiation and the regulation of myeloid cell differentiation. Within the CC category, prominent associations were observed with elements such as the cytosolic ribosome and ribosomal subunit. Notably, the molecular function (MF) category revealed significant enrichment in structural constituents of the ribosome, RAGE receptor and ubiquitin protein ligase binding for the DEGs. The results of the KEGG analysis (Figure S2C) underscored the robust association of genes with the regulation of hemopoiesis, myeloid cell differentiation, the regulation of myeloid cell differentiation and the homeostasis of the number of cells. Notably, KEGG mapping represents a predictive approach that reconstructs molecular network systems on the basis of functional orthologues derived from molecular building blocks. The enrichment analysis revealed that the DEGs were involved mainly in cell differentiation and were related to cytoplasmic ribosomes. This finding also indicates the important role of DEGs in AS cell differentiation.

### 3.3 CD74 and JUN as Hub Genes in Immune Cells

**FIGURE 3:**
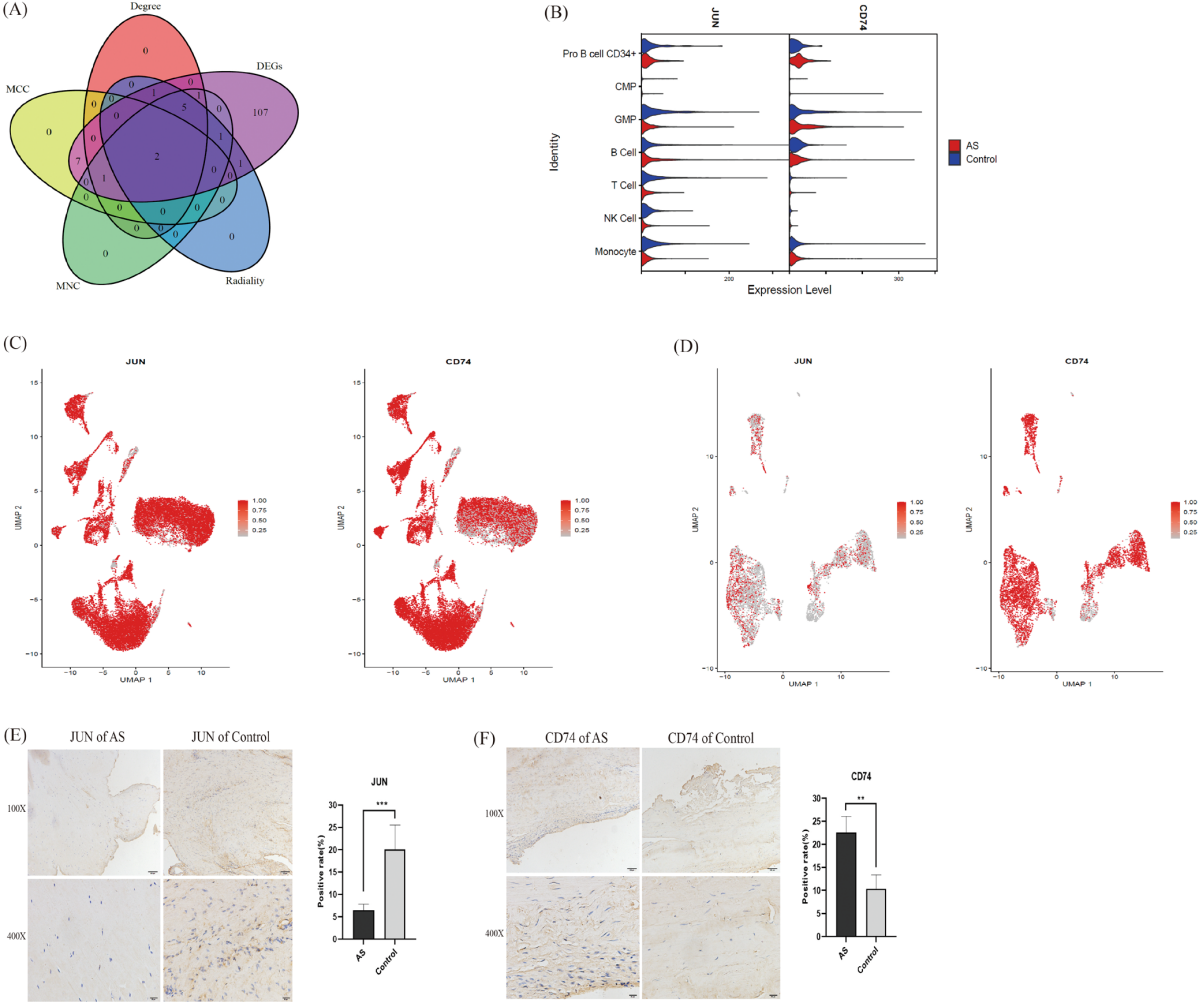
Expression and validation of genes CD74 and JUN in AS. (A) CD74 and JUN identified as core genes through the intersection of four Cytoscape algorithms. (B) Violin plots showing CD74 and JUN expressions across different cell clusters. (C) UMAP plots of CD74 and JUN expressions in the experimental group, with darker colours indicating higher expression. (D) UMAP plots of CD74 and JUN expressions in the validation group. (E, F) Immunohistochemistry results show lower JUN expression and higher CD74 expression in AS samples compared to the control group. ‘*’ indicates *p* < 0.05, ‘**’ indicates *p* < 0.01 and ‘***’ indicates *p* < 0.001.

We utilised the STRING online tool to construct PPI networks (Figure S1G) for the DEGs from the single‐cell sequencing data. Using Perl and Cytoscape, we constructed a network that identified 34 upregulated and 50 downregulated genes, as visualised in Figure S2D. Four algorithms within the Cytoscape‐Hubba plug‐in were used to select the top 10 core genes for each (Figure S2E–H). The intersection with DEGs revealed two core genes: CD74 (upregulated) and JUN (downregulated) (Figure 3A). Violin plots revealed the expression of CD74 and JUN across different cell types (Figure 3B). UMAP plots of the cell annotation results for both the experimental and validation groups revealed high CD74 expression in both groups, whereas JUN was expressed primarily in the experimental group (Figure 3C,D). In addition, to compare with the single‐cell results of AS, we analysed single‐cell sequencing data from three control samples, obtaining a total of 24 clusters (Figure S3A). Through cell annotation, we identified nine cell types (Figure S3B). The expression levels of CD74 and JUN in various cell types within the control group were visualised using UMAP plots (Figure S3C). We observed that both genes were expressed in different cells within the control group. To quantify and further validate the expression of CD74 and JUN in AS, we performed immunohistochemical analysis on interspinous ligament samples from AS patients and controls. CD74 was significantly upregulated, and JUN was downregulated in the AS group (*p* < 0.05) (Figure 3E,F).

### 3.4 T and NK Cells Are Key Immune Cells in AS

**FIGURE 4:**
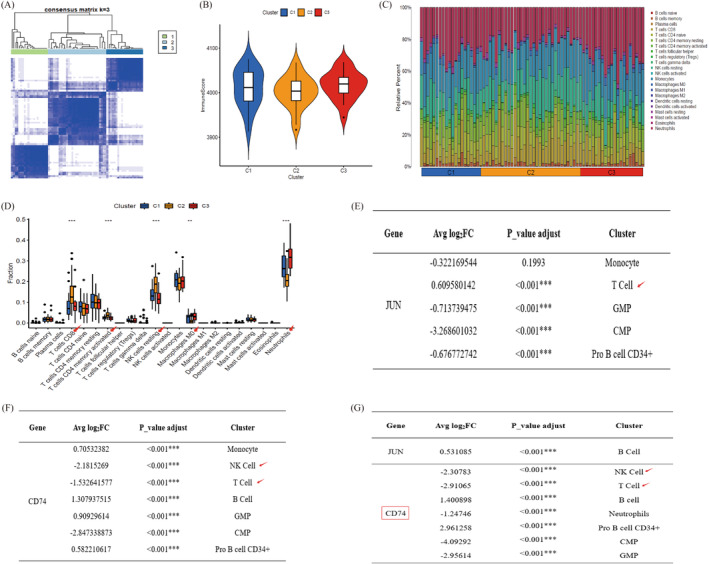
Immune cell infiltration and gene expression in AS clusters. (A) Pseudo‐time series and GSE73754 analysis identified three optimal clusters. (B) Immune scores evaluated for the three clusters. (C) Immune cell proportions across the clusters, showing uniform distribution. (D) Immune cell infiltration analysis revealed significant differences among T cells, NK cells, macrophages and neutrophils. (E, F) Expression of JUN and CD74 in annotated cells of the experimental group. (G) Expression of JUN and CD74 in annotated cells of the validation group. Comprehensive analysis highlights CD74 as the hub gene, with T cells and NK cells identified as key immune cells. ‘**’ indicates *p* < 0.01 and ‘***’ indicates *p* < 0.001.

Using the GSE73754 dataset and pseudo‐time series analysis, we identified branching genes and divided the co‐expressed genes into three distinct clusters (Figure 4A). After assessing the immune efficacy of these clusters, we observed minimal differences in immune scores among them (Figure 4B). Next, immune infiltration analysis revealed statistically significant differences in T cells, NK cells, macrophages and neutrophils (Figure 4C,D). These results highlighted that the immune status varied across gene subtypes and loci. To further validate the differences in the expression of the JUN and CD74 genes in different cells, we analysed their expression across cell types. In the experimental group, JUN expression in T cells and CD74 expression in T cells and NK cells were significantly different (Figure 4E,F). In the validation group, JUN was significantly expressed in B cells, whereas CD74 was significantly expressed in both T cells and NK cells (Figure 4G). Based on the above findings, we believe that T cells and NK cells may have more obvious effects on AS and that CD74 can be used as a key target gene.

### 3.5 T and NK Cells Are Associated With Other Cell Types in AS

**FIGURE 5:**
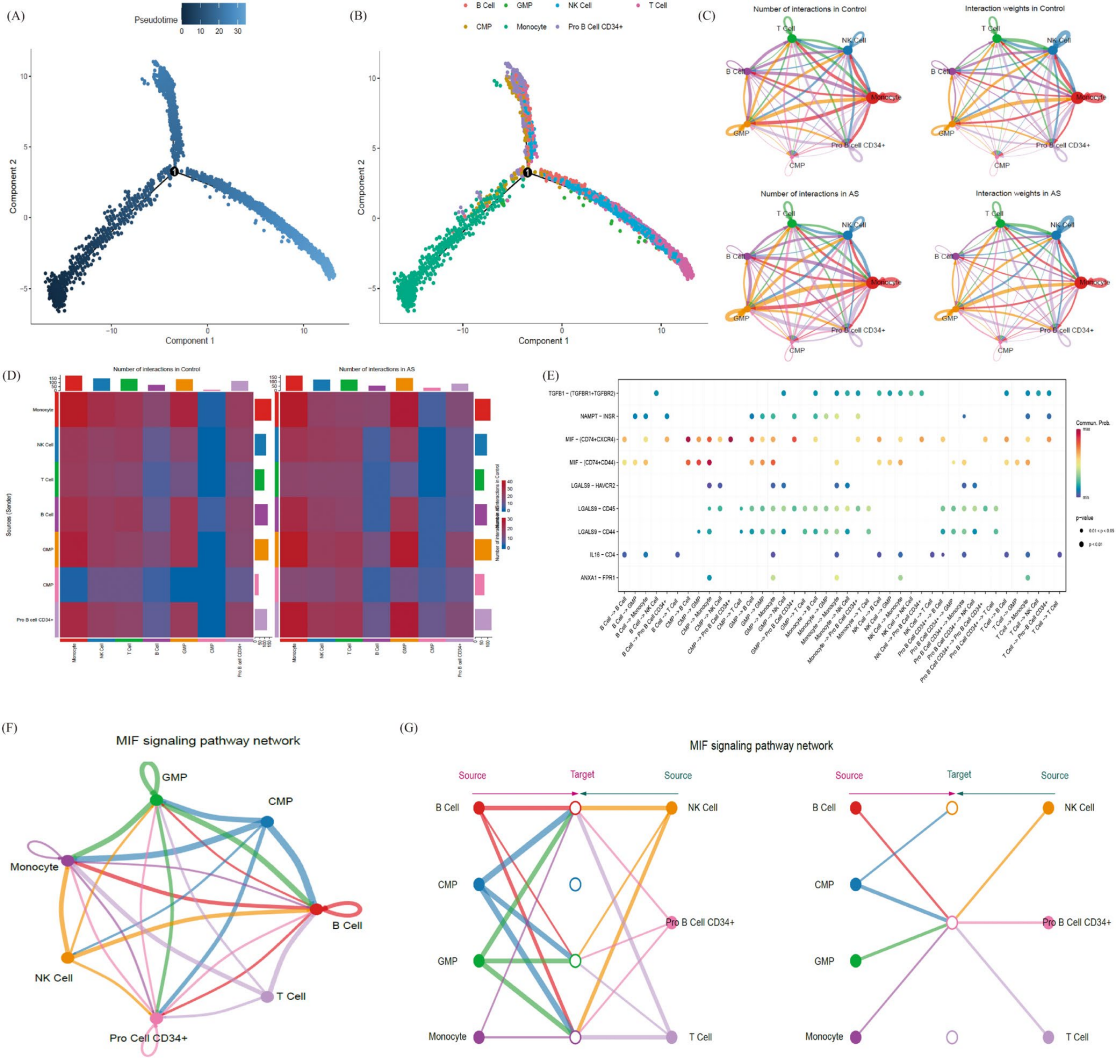
Intercellular communication and MIF signalling in AS. (A) Pseudo‐temporal analysis of single cells, where darker colours represent earlier time points. (B) Distribution of different cell types in the pseudo‐temporal analysis. (C) Shell plot showing the number and strength of intercellular communications in the experimental group between AS and control groups. (D) Heat map comparing the intercellular communication between AS and control groups. (E) Key signalling pathways involved in cell interactions, where circle size represents the significance of the association and colour indicates the strength. (F) Cell interactions within the MIF signalling pathway. (G) Hierarchical diagram illustrating paracrine and autocrine relationships among cell types within the MIF signalling pathway.

The pathological progression of AS involves complex interactions between various cell types. Pseudo‐temporal analysis revealed significant heterogeneity among the cell populations, with T/NK cells, B cells and myeloid cells clustering in three distinct branches (Figure 5A,B). To explore cell interactions further, we used the ‘Cell Chat’ R package, which focuses on receptor–ligand pairs involved in paracrine/autocrine signalling, extracellular matrix receptor interactions and intercellular contact. A comparison of the number and intensity of intercellular interactions between the control and AS groups revealed altered interactions for T and NK cells in AS, as shown in the shell and heatmaps (Figure 5C,D). Key signalling pathways, including the macrophage migration inhibitory factor (MIF) pathway, were notably expressed in AS (Figure 5E), including the CD74/CXCR4 and CD74/CD44 receptor–ligand pairs. Further analysis of the MIF pathway indicated that T and NK cells interact with other cells primarily through paracrine signalling, whereas monocytes and B cells engage in both autocrine and paracrine signalling (Figure 5F,G).

**FIGURE 6:**
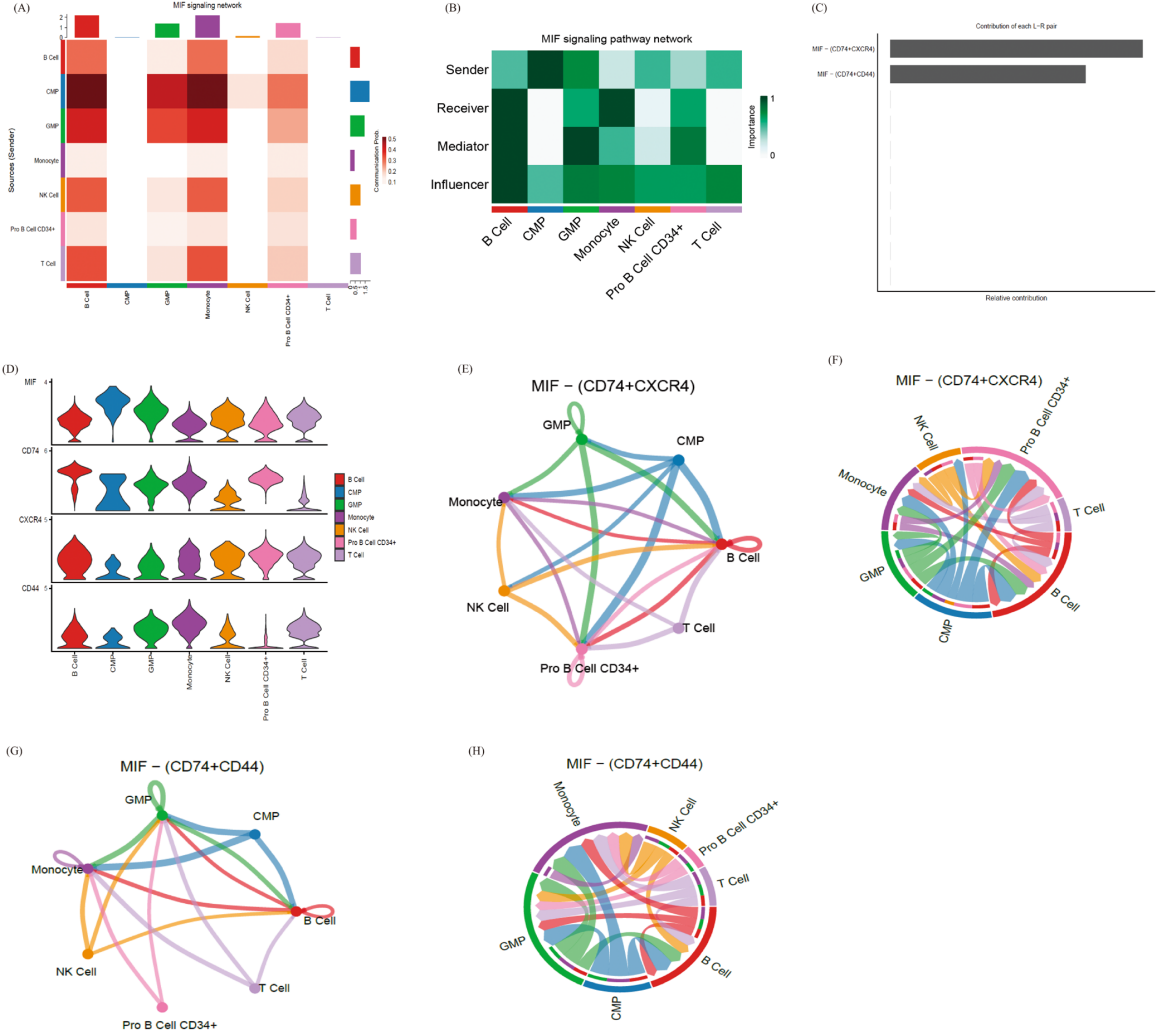
Analysis of cell interactions via the MIF signalling pathway in AS. (A) Heat map showing cell‐type interactions in the MIF signalling pathway, where the horizontal axis represents receptor cells and the vertical axis represents ligand cells. Redder colours indicate stronger interactions. (B) Plot showing cell roles (sender, receiver, mediator, influencer) on the horizontal axis, with darker colours indicating higher significance. (C) Ligand–receptor pair contributions to the MIF signalling pathway, highlighting the significant roles of CD74/CXCR4 and CD74/CD44 pairs. (D) Violin plots illustrating the receptor and ligand gene expression in each cell type. (E, G) Network diagrams depicting the interactions of cells involved in the CD74/CXCR4 and CD74/CD44 receptor complexes. (F, H) Chord diagrams illustrating the interactions between cells in the CD74/CXCR4 and CD74/CD44 receptor complexes.

To further elucidate the specific role of various types of cells in the MIF signalling pathway, our investigation highlighted the significant role of the MIF signalling pathway in orchestrating communication among diverse immune cells (Figure 6A). In addition to the powerful signalling function of MIF, in this pathway, various types of cells can act as signal receptors, bridges or information conduits, potentially influencing other cells (Figure 6B). Notably, CD74−CXCR4 and CD74−CD44 were found to be prominent receptor complexes within the MIF signalling pathway (Figure 6C). Examination of the expression of CD74, CD44 and CXCR4 across different cell types revealed their presence in various cells (Figure 6D). Further scrutiny of the communication interactions facilitated by CD74−CXCR4 and CD74−CD44 indicated that T cells, NK cells and monocytes, which exhibit significant differences, may contribute to the differentiation of AS cells as either receptor cells or ligands (Figure 6E–H). Our findings indicate that the immune cell interaction of AS occurs mainly through the MIF signalling pathway, among which the expression of CD74, CD44 and CXCR4 is the most important.

## 4 Discussion

Ankylosing spondylitis manifests as a classic inflammatory disease, typically initiating inflammation in the sacroiliac (SI) joint and progressing to spinal fusion, resulting in the characteristic ‘bamboo spine’. As a subtype of spondyloarthritis (SpA), AS triggers inflammation not only in the spine but also in peripheral joints, ligaments and tendons [23, 24]. The specific pathogenic mechanisms of AS remain incompletely understood, and several hypotheses have been proposed. These include investigations into arthritogenic peptides and the potential for molecular mimicry between foreign and self‐peptides. Despite evidence suggesting T‐cell clonal expansion in patients, identifying the antigens responsible for initiating autoinflammation in AS remains challenging [25, 26]. In AS, neutrophils and macrophages/monocytes exhibit characteristic expansion, leading to synovial infiltration. Haematological analyses consistently revealed elevated ratios of neutrophils to lymphocytes (NLR), platelets to lymphocytes (PLR) and monocytes to lymphocytes (MLR) in patients with AS than in those without AS. These haematological changes signify an altered immune profile in patients with AS, suggesting a potential role for these immune cells in the pathogenesis of the disease [27, 28]. NK cells, crucial players in innate immunity with roles in both innate and adaptive immune responses, are also implicated in AS pathogenesis [29]. This study elucidates the pivotal involvement of key immune cells—T cells, NK cells and monocytes—in the immune response to AS. In particular, T cells, crucial components of adaptive immune defence, dynamically reprogramme their metabolic needs in response to environmental cues. Ineffective adaptation of T cells to specific metabolic requirements can result in suboptimal cellular responses, leading to compromised immune function or, conversely, excessive activity, leading to autoimmune tissue damage [30]. T cells, which are responsible for initiating and regulating the body's response to infections, significantly influence the development of AS [31, 32]. Importantly, CD4+ and CD8+ T cells, each of which possesses distinct regulatory features in the immune response, were identified as being activated in AS through immune cell infiltration analysis and single‐cell sequencing, indicating their likely participation in the immune reaction processes associated with AS [10]. NK cells, a subset of large granular lymphocytes, constitute integral components of innate immunity, bridging the gap between innate and adaptive immune responses. Through their cytokines and cytotoxic functions, NK cells play crucial roles in regulating immune responses and are implicated in the pathogenesis of various immune‐mediated diseases, including AS, Behcet's disease, multiple sclerosis and rheumatoid arthritis [33, 34]. Monocyte and macrophage subpopulations play essential roles in immune regulation in both cancer immunotherapy and inflammatory immunotherapy. In the immune system, macrophages typically develop and differentiate from monocytes [35, 36, 37]. Our preliminary study revealed that monocytes play a crucial immune role in AS, and LYN has been identified as a potential immunotherapeutic target [38].

CD74 is a nonpolymorphic type II transmembrane glycoprotein that has multiple biological functions in physiological and pathological situations in addition to being a chaperone of MHC class II molecules [39]. CD74 acts as a high‐affinity receptor for macrophage migration inhibitor (MIF) [40, 41], which regulates T‐ and B‐cell development, dendritic cell (DC) movement, macrophage inflammation and thymus selection. Abdelaziz MM et al. reported that the positive rate of anti‐CD74 IgG antibodies was significantly increased in axSpA patients, and there was a significant difference in the positive rate of anti‐CD74 IgG antibodies between positive and negative patients with disease activity [42]. Baerlecken et al. reported that anti‐CD74 antibodies can provide an important additional tool for the diagnosis of SpA [43]. Similarly, several studies have shown that IgA anti‐CD74 may play an important role in the pathogenesis of AS [44, 45]. Through the meticulous screening of core genes via single‐cell sequencing technology, we reaffirmed the crucial role of CD74. Immunohistochemical experiments and analysis of intercellular communication provided further insights into the importance of CD74 in AS.

Through pseudo‐time series analysis, immune infiltration analysis and intercellular communication analysis, our observations revealed that T cells, NK cells and monocytes are important immune cells in AS. The intercellular MIF signalling pathway may emerge as a significant influencer in the differentiation process of AS cells and is mediated primarily by the receptor complexes CD74−CXCR4 and CD74−CD44. Macrophage migration inhibition factor, a cytokine with crucial roles in development, metabolism and immune responses [46, 47, 48], effectively inhibits AS progression in animal models, underscoring its importance in AS pathogenesis [49]. CD74 acts as an MIF receptor, whereas CD44, CXCR2 and CXCR4 function as coreceptors. The binding of MIF to CD74 induces a lateral shift in the coreceptor, causing serine phosphorylation of the intracellular region of CD74 [50]. The coreceptor‐mediated effects of CXCR2 and CXCR4 contribute to the recruitment of neutrophils, macrophages and lymphocytes.

In summary, the present study identified and validated the hub gene CD74, utilising various analytical methods based on single‐cell sequencing data from both AS and non‐AS samples combined with GSE datasets. Cell communication analysis revealed temporal differences in the differentiation stages of different immune cells in AS. The MIF signalling pathway may emerge a crucial player in AS immune cells, and its interaction is likely mediated through receptor complexes, particularly CD74−CXCR4 and CD74−CD44. However, it is important to note that this study has limitations, such as a relatively small sample size and a limited number of experiments. Consequently, the findings are currently in the theoretical stage, and further validation through specific experiments is warranted in future studies.

## 5 Conclusion

This study thoroughly analysed single‐cell sequencing data obtained from both AS and non‐AS samples validated with external datasets, revealing the importance of T and NK cells in AS, and revealed that CD74 may be a potential key target. This study extensively explored pivotal immune cells in AS, elucidating specific interactions among them, particularly highlighting the involvement of the MIF signalling pathway, as evidenced by cell communication analysis. These findings increase our understanding of the immune mechanisms underlying AS and provide potential novel targets for therapeutic interventions.

## 6 Limitations

In this study, through a comprehensive analysis of single‐cell sequencing data and transcriptome data, important immune cells and DEGs in AS were identified, and the interconnections among various immune cells in AS were explained through cell communication analysis. However, it was only experimentally verified by immunohistochemistry, which is a limitation of our study. In a follow‐up study, we will elaborate on the specific role of immune cells in the pathological process of AS in more detail through more comprehensive experimental design and development. Second, we included a small single‐cell sample size, and we hope to further support our findings with a larger sample size in the future.

## Author Contributions

**Tianyou Chen:** conceptualization (equal), methodology (equal), writing – review and editing (equal). **Shengyu Ning:** methodology (equal), writing – original draft (equal). **Jichong Zhu:** validation (equal). **Xinli Zhan:** supervision (equal). **Chenxing Zhou:** formal analysis (equal), investigation (equal). **Chengqian Huang:** writing – original draft (equal). **Shaofeng Wu:** resources (equal). **Bin Zhang:** data curation (equal). **Sitan Feng:** software (equal). **Jiarui Chen:** validation (equal). **Jiang Xue:** visualization (equal). **Zhenwei Yang:** data curation (equal). **Chong Liu:** conceptualization (equal), funding acquisition (equal), supervision (equal), writing – review and editing (equal).

## Ethics Statement

The study was approved by the ethics review board of our institution (Approval No. 2024‐E041‐01) in accordance with the Declaration of Helsinki. All methods were carried out in accordance with relevant guidelines and regulations. Written informed consent was obtained from all individual patients included in the study.

## Consent

All authors have reviewed the manuscript and agreed to submit it for publication.

## Conflicts of Interest

The authors declare no conflicts of interest.

## Supporting information


**Figure S1** Data distribution and gene expression in experimental and validation groups. (A) Overview of the data in the experimental group. (B) Distribution of six samples in the experimental group after batch effect removal with Harmony. (C) Overview of the data in the validation group. (D) Distribution of two AS samples in the validation group after batch effect removal with Harmony. (E) Heat maps displaying the top five differentially expressed genes in each cell type in the experimental group. (F) Heat maps displaying the top five differentially expressed genes in each cell type in the validation group. (G) The PPI network constructed using the STRING online tool.

**Figure S2** Differential genes play a significant role in AS. (A/B) Graphical representation of GO enrichment analysis results for DEGs. (C) KEGG analysis outcomes for DEGs. (D) Visualise upregulated genes (red) and downregulated genes (green) via Cytoscape. Panel (E) shows the 10 hub genes obtained via the radiality algorithm. Panel (F) shows the 10 hub genes obtained via the degree algorithm. Panel (G) shows the 10 hub genes obtained via the MCC algorithm. Panel (H) shows the 10 hub genes obtained via the MNC algorithm.

**Figure S3** Identification of cell clusters and CD74/JUN expression patterns in control group. (A) Twenty‐four clusters identified in the control group. (B) Nine cell types identified in the control group after annotation. (C) UMAP plots of CD74 and JUN expressions in the control group, with darker colours indicating higher expression.

**Table S1** The DEGs were discerned from the single‐cell sequencing data when comparing the AS group with the non‐AS group.

**Table S2** The GO analysis results of DEGs.

**Table S3** The KEGG analysis results of DEGs.

**Table S4** The basic information of patients included in immunohistochemistry.

Appendix S1

## Data Availability

The datasets used and/or analysed during the current study are available from the corresponding author on reasonable request.
