# Supplementary figures and images for: Exploring T Cell and NK Cell Involvement in Ankylosing Spondylitis Through Single‐Cell Sequencing
Source: J Cell Mol Med. 2024 Dec 16;28(24):e70206. doi: 10.1111/jcmm.70206 (PMC11648971; doi:10.1111/jcmm.70206)

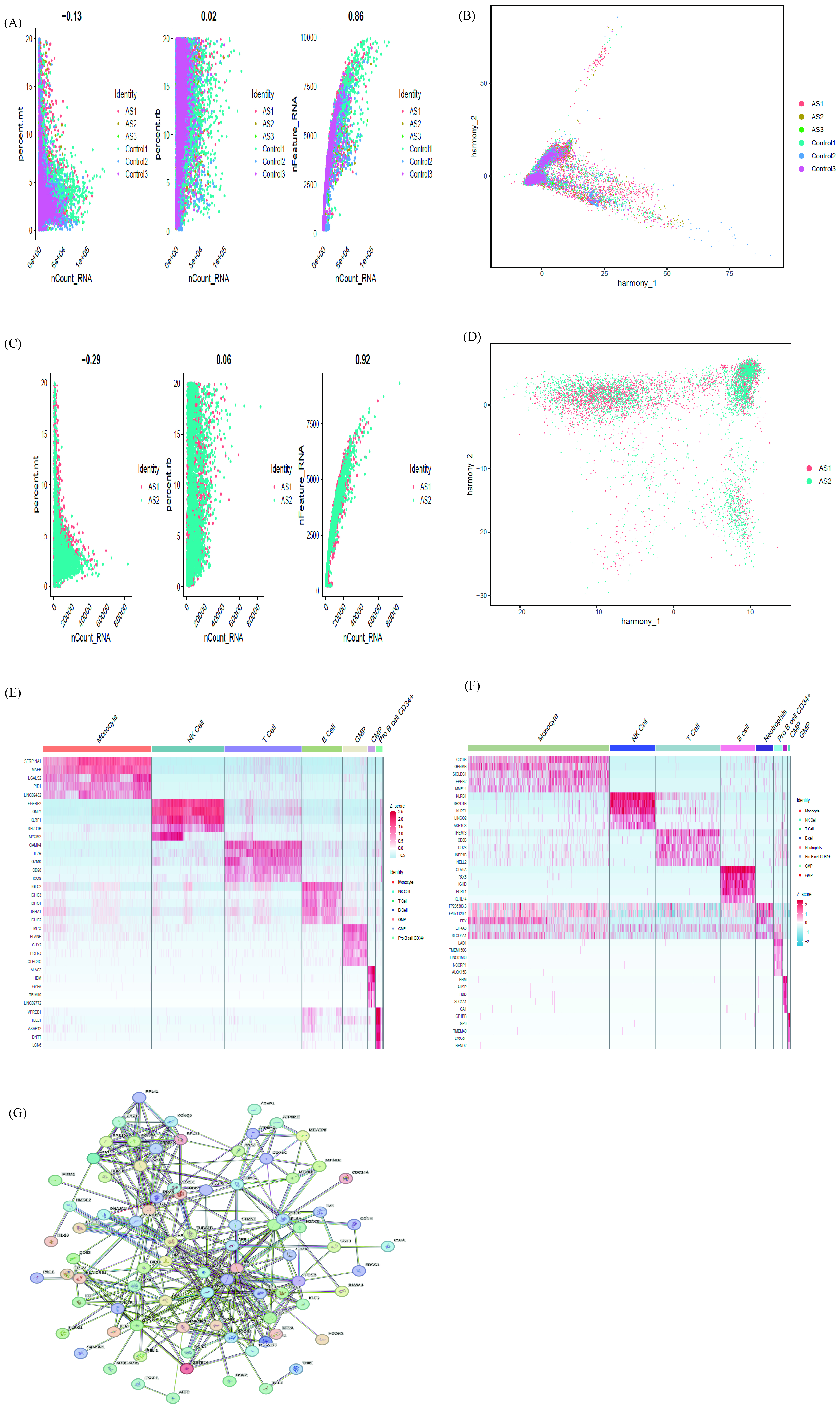

Supplement: Supplementary file 1 — Figure S1 Data distribution and gene expression in experimental and validation groups. (A) Overview of the data in the experimental group. (B) Distribution of six samples in the experimental group after batch effect removal with Harmony. (C) Overview of the data in the validation group. (D) Distribution of two AS samples in the validation group after batch effect removal with Harmony. (E) Heat maps displaying the top five differentially expressed genes in each cell type in the experimental group. (F) Heat maps displaying the top five differentially expressed genes in each cell type in the validation group. (G) The PPI network constructed using the STRING online tool. [file JCMM-28-e70206-s002.jpg]

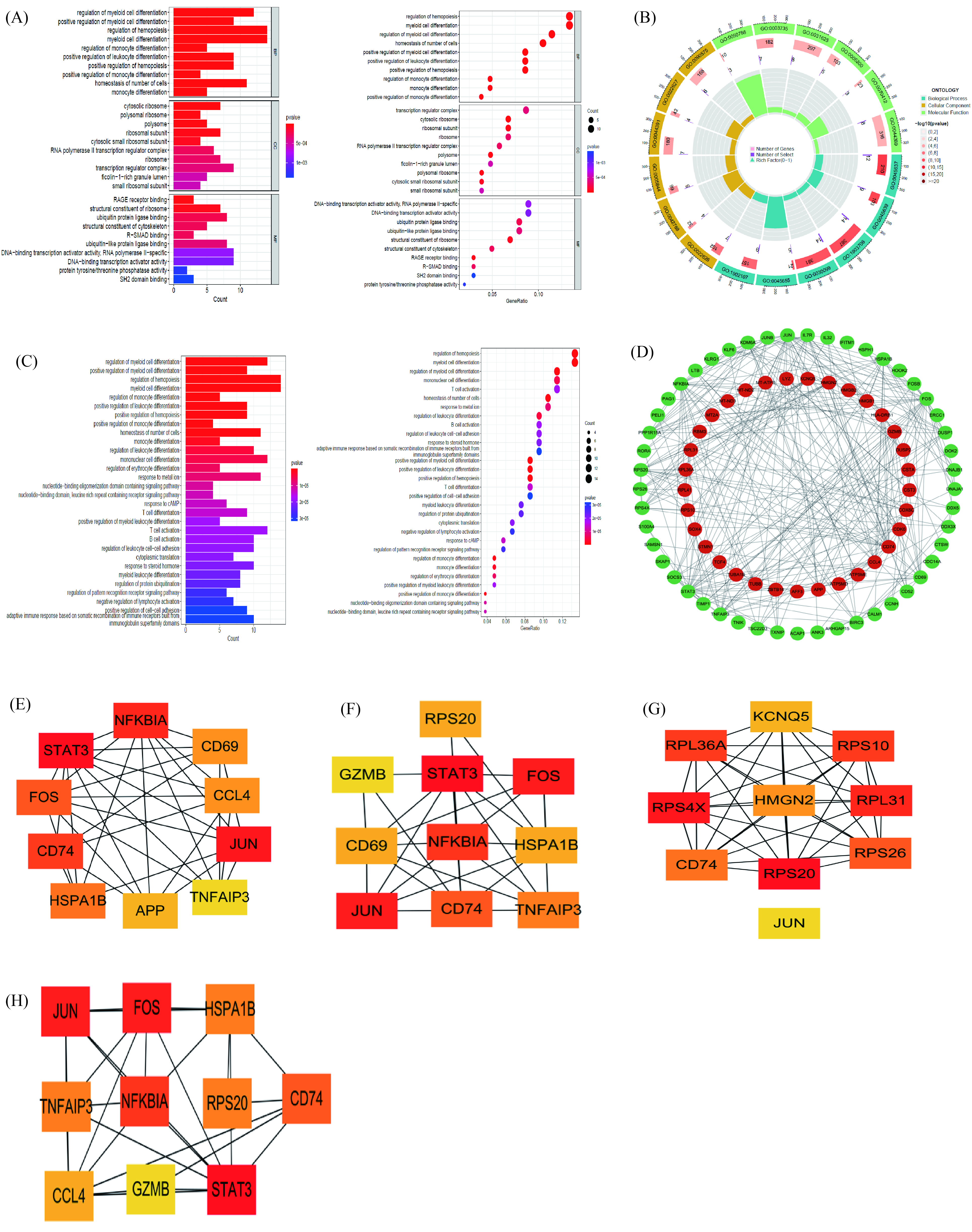

Supplement: Supplementary file 2 — Figure S2 Differential genes play a significant role in AS. (A/B) Graphical representation of GO enrichment analysis results for DEGs. (C) KEGG analysis outcomes for DEGs. (D) Visualise upregulated genes (red) and downregulated genes (green) via Cytoscape. Panel (E) shows the 10 hub genes obtained via the radiality algorithm. Panel (F) shows the 10 hub genes obtained via the degree algorithm. Panel (G) shows the 10 hub genes obtained via the MCC algorithm. Panel (H) shows the 10 hub genes obtained via the MNC algorithm. [file JCMM-28-e70206-s001.jpg]

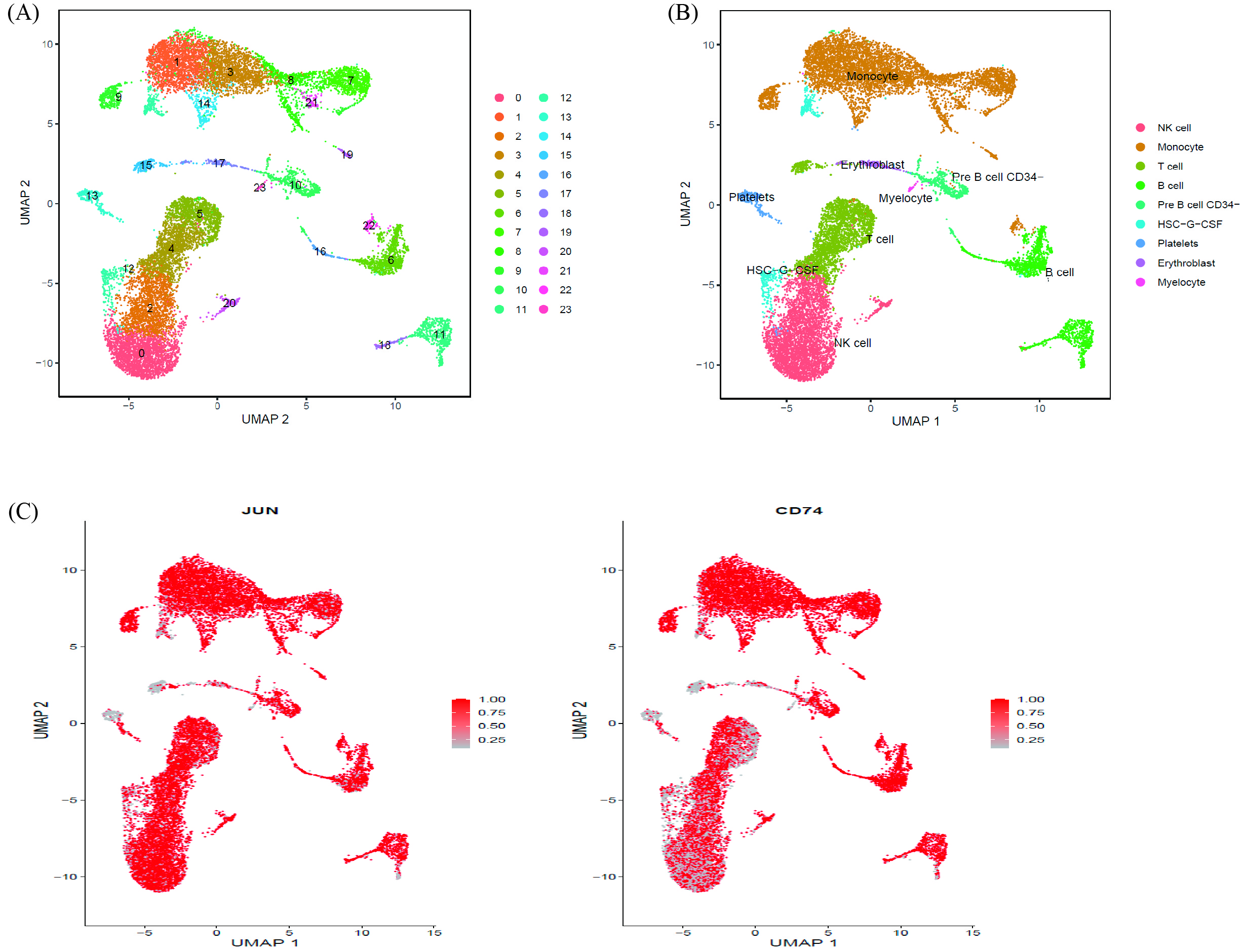

Supplement: Supplementary file 3 — Figure S3 Identification of cell clusters and CD74/JUN expression patterns in control group. (A) Twenty‐four clusters identified in the control group. (B) Nine cell types identified in the control group after annotation. (C) UMAP plots of CD74 and JUN expressions in the control group, with darker colours indicating higher expression. [file JCMM-28-e70206-s006.jpg]
