# Supplementary material for: Exploring T Cell and NK Cell Involvement in Ankylosing Spondylitis Through Single‐Cell Sequencing
Source: J Cell Mol Med. 2024 Dec 16;28(24):e70206. doi: 10.1111/jcmm.70206 (PMC11648971; doi:10.1111/jcmm.70206)
Supplement: Supplementary file 7 — Table S4 The basic information of patients included in immunohistochemistry. [file JCMM-28-e70206-s003.docx]

Supplement Table 4 Patients' baseline information of the immunohistochemical analysis.

| Patients | Diagnosis | Gender | Age(years) | Height(cm) | Weight(kg) | BMI |
| --- | --- | --- | --- | --- | --- | --- |
| N1 | Spinal fracture | Male | 32 | 168 | 73 | 25.9 |
| N2 | Spinal fracture | Male | 37 | 175 | 81 | 26.4 |
| N3 | Spinal fracture | Male | 41 | 182 | 85 | 25.7 |
| N4 | Spinal fracture | Male | 28 | 164 | 65 | 24.2 |
| AS1 | AS with kyphosis | Male | 33 | 167 | 51 | 18.3 |
| AS2 | AS with kyphosis | Male | 26 | 173 | 55 | 18.4 |
| AS3 | AS with kyphosis | Male | 43 | 159 | 48 | 19.0 |
| AS4 | AS with kyphosis | Male | 38 | 164 | 53 | 19.7 |

BMI：Body Mass Index.
