# Supplementary material for: Exploring T Cell and NK Cell Involvement in Ankylosing Spondylitis Through Single‐Cell Sequencing
Source: J Cell Mol Med. 2024 Dec 16;28(24):e70206. doi: 10.1111/jcmm.70206 (PMC11648971; doi:10.1111/jcmm.70206)
Supplement: Supplementary file 8 — Appendix S1 [file JCMM-28-e70206-s007.docx]

**Supplementary Material**

**1 Clinical data of patients**

The inclusion criteria were as follows: (1) Patients who met the modified New York AS criteria. (2) Good compliance, no serious cardiovascular and cerebrovascular history. (3) The patient voluntarily underwent a blood routine examination. The exclusion criteria were as follows: (1) Patients who could not tolerate blood drawing or had coagulation dysfunction. (2) Patients with body temperature exceeding 37.3 °C upon admission. The inclusion criteria of the control group (patients without AS) were as follows: (1) diagnosed as non-AS patients. (2) Good compliance, no history of serious cardiovascular and cerebrovascular diseases. (3) Patients who voluntarily underwent blood routine examination. The exclusion criteria were as follows: (1) Patients who could not tolerate blood drawing or had coagulation dysfunction. (2) Patients with a body temperature greater than 37.3 °C upon admission.

**2 Single-Cell Library Construction**

The samples employed for single cell sequencing emanated from spinal vertebral bone marrow blood derived from patients undergoing surgical procedures. This cohort encompassed three patients diagnosed with AS. Fresh specimens, harvested during surgical resection, were promptly placed in MACS tissue storage solution (Miltenyi Biotec, Germany), and expedited to our laboratory without delay. The inclusion criteria were as follows: (1) Patients who were diagnosed with AS following the New York criteria. (2) Presence of thoracolumbar vertebra or lumbar kyphosis. (3) Clear indication of surgical correction and willingness to undergo surgery. The exclusion criteria were as follows: (1) Other connective tissue diseases, such as rheumatoid arthritis or systemic lupus erythematosus, were present. (2) Clear contraindications to corrective surgery or not willing to undergo corrective surgery. The inclusion criteria for the control group were as follows: (1) Lumbar/thoracic vertebral fractures were confirmed by history and imaging examinations. (2) A clear indication of surgical correction and willingness to undergo surgery. The exclusion criteria were as follows: (1) Other connective tissue diseases, such as rheumatoid arthritis or systemic lupus erythematosus, were present. (2) There were clear contraindications to surgery, or patients were not willing to undergo surgery. Fresh specimens collected at the time of surgical excision were immediately stored in MACS tissue storage solution (Miltenyi Biotec, Germany) and transported to the laboratory.

**2.1 Single-Cell Library Construction**

Firstly, the single-cell suspension was obtained, and its activity was tested. The cell activity was >85% and the monodispersity was good and the impurity content was low. Then the microfluidic channel oil-in-water droplet library was prepared and operated on the machine. In order to ensure the effective capture efficiency of single cells, the cell concentration is generally required to be controlled within the range of 700-1200cell/uL. In the process of library preparation, a single Gel Bead and a single cell were coated with a single oil-coated water drop to form a GEM, and each Gel Bead has a unique Barcode and UMI sequence and Poly-dT primer sequence for initiating reverse transcription reaction. Subsequently, in this GEM reaction system, the cells are broken and lysed to release mRNA Full cDNA was generated by reverse transcription reaction with Poly-dT primer sequence under the action of reverse transcriptase. The cDNA was then amplified and the library was constructed.

**2.2 Processing of scRNA-Sequencing Data**

After the completion of the library construction, Qubit3.0 was used for preliminary quantification, and the library was diluted to 1ng/uL. Then Agilent 2100 was used to detect the insert size of the library. After the insert size met the expectation, The StepOnePlus Real-Time PCR System fluorescent quantitative PCR instrument was used for Q-PCR, and the effective concentration of the library was accurately quantified (the effective concentration index of the library was not less than 10nM) to ensure the quality of the library. Qualified libraries were sequenced using Illumina platform. Raw reads were obtained. First, data were intercepted according to the 10x transcriptome library structure, and Read1 and target Read2 containing arcode and UMI sequence information were obtained. Then Cell Range, the official 10x software, was used for data analysis and processing.
